# Potential Sexual Transmission of Antifungal-Resistant *Trichophyton indotineae*

**DOI:** 10.3201/eid3004.240115

**Published:** 2024-04

**Authors:** Stephanie Spivack, Jeremy A.W. Gold, Shawn R. Lockhart, Priyanka Anand, Laura A.S. Quilter, Dallas J. Smith, Briana Bowen, Jane M. Gould, Ahmed Eltokhy, Ahmed Gamal, Mauricio Retuerto, Thomas S. McCormick, Mahmoud A. Ghannoum

**Affiliations:** Temple University Hospital Section of Infectious Diseases, Philadelphia, Pennsylvania, USA (S. Spivack);; Centers for Disease Control and Prevention, Atlanta, Georgia, USA (J.A.W. Gold, S.R. Lockhart, P. Anand, L.A.S. Quilter, D.J. Smith);; Department of Public Health, Philadelphia (B. Bowen, J.M. Gould);; Center for Medical Mycology, Case Western Reserve University and University Hospitals Cleveland Medical Center, Cleveland, Ohio, USA (A. Eltokhy, A. Gamal, M. Retuerto, T.S. McCormick, M.A. Ghannoum)

**Keywords:** tinea, fungi, *Trichophyton indotineae*, sexually transmitted infections, dermatophytes, dermatomycoses, antifungal agents, antifungal resistance, antimicrobial resistance, terbinafine, itraconazole, fluconazole, antimicrobial stewardship, Pennsylvania, United States

## Abstract

We describe a case of tinea genitalis in an immunocompetent woman in Pennsylvania, USA. Infection was caused by *Trichophyton indotineae* potentially acquired through sexual contact. The fungus was resistant to terbinafine (first-line antifungal) but improved with itraconazole. Clinicians should be aware of *T. indotineae* as a potential cause of antifungal-resistant genital lesions.

Dermatophytosis, also called ringworm or tinea, is a common superficial fungal skin infection most often caused by *Trichophyton*, *Microsporum,* or *Epidermophyton* fungi and often treated using over-the-counter topical antifungal agents (*1*). Oral terbinafine is a first-line antifungal treatment for extensive skin infections, which typically occur in immunocompromised or older persons (*1*). Outbreaks of extensive, recalcitrant, and frequently terbinafine-resistant dermatophytosis in immunocompetent persons are ongoing in southern Asia because of the recently emerged dermatophyte *Trichophyton indotineae* (formerly *Trichophyton mentagrophytes* genotype VIII). *T. indotineae* typically causes tinea faciei, corporis, or cruris; easily spreads person-to-person; and has been reported globally, including in multiple US states (*2*–*4*). Laboratory identification requires advanced molecular techniques because culture-based methods cannot distinguish *T. indotineae* from other *Trichophyton* species (*2*). 

Previous reports describe sexual transmission of genital dermatophytosis (*5*,*6*), including cases caused by *T. mentagrophytes* genotype VII, a dermatophyte closely related to *T. indotineae* but not associated with terbinafine resistance (*7*,*8*). We report a case of tinea genitalis in an immunocompetent woman in Pennsylvania, USA, that was caused by an antifungal-resistant *T. indotineae* strain potentially acquired through sexual contact. Our study was reviewed by the Centers for Disease Control and Prevention (CDC) and conducted consistent with applicable federal laws and CDC policy. 

During winter 2022, a healthy young cisgender woman traveled to South Asia. While there, she had vaginal intercourse with a man who had purple genital and buttocks lesions. Subsequently, she experienced similar lesions, beginning on her inner thigh, then spreading to her genitals and buttocks. In spring 2022, she returned to the United States and sought care from a primary care provider and dermatologist. She received mometasone 0.1% ointment (topical medium-potency corticosteroid) for suspected contact dermatitis, econazole 1% (topical antifungal) cream, a prednisone taper pack, and diphenhydramine. The reported lesions did not resolve, and corticosteroids worsened the condition. The result of a thigh skin-punch biopsy was positive for hyphae by periodic acid-Schiff stain, consistent with dermatophytosis. The patient subsequently received multiple antifungal courses including topical ketoconazole, oral terbinafine (250 mg/d for 2 weeks), and fluconazole (150 mg/wk to 200 mg/d for >20 cumulative weeks), all without lesion resolution. 

In spring 2023, physical examination by an infectious disease physician revealed an annular, scaling, hyperpigmented eruption on the patient’s buttocks involving the intergluteal cleft and 3 small hyperpigmented areas on the mons pubis. She reported having a new male sexual partner in the United States who developed similar lesions on his genitals after they had sexual intercourse. Given clinical suspicion for *T. indotineae* infection, she was prescribed a 1-week course of itraconazole. On telephone follow-up 4 days later, she reported decreasing rash size and pruritis. She missed a follow-up visit but was present for a telemedicine visit 6 weeks later, when she reported resumption of pruritis and 2 new small pruritic patches on her right buttock and labia. She was prescribed an additional 2-week course of itraconazole (200 mg 2×/d). Her pruritis resolved, and she reported no recurrence at 3-months follow-up. The rash on the patient’s sexual partner resolved, but we are unaware of the treatment he received. 

The infectious disease physician sent a skin scraping from the gluteal region for fungal culture and advanced mycologic testing at the Center for Medical Mycology of the University Hospitals Cleveland Medical Center (Cleveland, OH, USA). Macroscopic and microscopic morphology and broth microdilution antifungal susceptibility testing (Figure) demonstrated MICs of 16 μg/mL for terbinafine, 16 μg/mL for fluconazole, 0.016 μg/mL for itraconazole, and *≤*0.031 μg/mL for efinaconazole (Clinical and Laboratory Standards Institute, https://clsi.org/standards/products/microbiology/documents/m38). Although breakpoints do not exist for dermatophytes, MIC ≥0.5 μg/mL for terbinafine has been correlated with resistance-conferring gene mutations (*4*). On the basis of internal transcribed spacer sequencing, we initially identified the isolate as *T. interdigitale*. Given concern for *T. indotineae* infection, we performed a BLAST search (https://blast.ncbi.nlm.nih.gov), which identified the isolate as *T. indotineae* (GenBank accession no. PP336547). 

**Figure:**
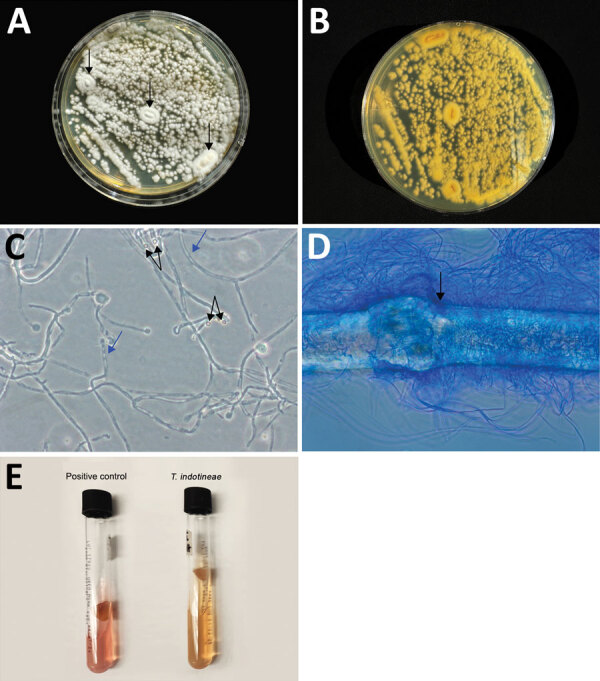
Results of gross and microscopic morphology and microbiological laboratory testing to identify *Trichophyton indotineae* in a woman in Pennsylvania, USA. A, B) Colonies were velvety white, flat, and had a raised center (arrow) (A) and a light yellow pigment on reverse (B). C) Numerous microconidia showed the pyriform and clavate forms (black arrow) and fungal hyphae with septation (blue arrows). Original magnification ×40. D) In vitro hair perforation test was positive (arrow). Original magnification ×100. E) *T. indotineae* had a negative urease test (yellowish color), while the positive control, *Trichophyton tonsurans*, was pinkish.

Our report highlights the emergence of antifungal-resistant *T. indotineae* as a cause of genital lesions and possible acquisition and transmission through sexual contact. Clinicians should be aware that visual inspection without diagnostic testing cannot reliably distinguish dermatophytosis from other causes of inflammatory skin conditions (e.g., contact dermatitis) (*9*). Subsequent inappropriate use of corticosteroids can exacerbate dermatophytosis. Diagnostic testing (e.g., with potassium hydroxide preparation) is essential to correctly diagnose and appropriately treat fungal skin infections (*1*,*9*). Increasing clinician awareness of dermatophytosis as a potential cause of genital lesions might prevent diagnostic delays (*7*). Itraconazole is often effective against *T. indotineae* infections, but there are challenges related to absorption, interactions between medications, insurance coverage, and possible need for prolonged therapy (sometimes requiring >3 months) and higher dosages of itraconazole (e.g., 200 mg 2×/d) (*2*,*10*). Strong inflammatory reactions that have been reported after initiation of antifungal treatment should not be confused with therapeutic failure (*6*). In conclusion, our report underscores the need for clinical vigilance, increased surveillance such as through sexual health provider networks to identify emerging trends in severe and antifungal-resistant dermatophytosis, studies to understand *T. indotineae* transmission dynamics, and laboratory capacity to identify dermatophyte species and test for antifungal susceptibility. 
